# Extreme absorption enhancement in ZnTe:O/ZnO intermediate band core-shell nanowires by interplay of dielectric resonance and plasmonic bowtie nanoantennas

**DOI:** 10.1038/s41598-017-07970-7

**Published:** 2017-08-08

**Authors:** Kui-Ying Nie, Jing Li, Xuanhu Chen, Yang Xu, Xuecou Tu, Fang-Fang Ren, Qingguo Du, Lan Fu, Lin Kang, Kun Tang, Shulin Gu, Rong Zhang, Peiheng Wu, Youdou Zheng, Hark Hoe Tan, Chennupati Jagadish, Jiandong Ye

**Affiliations:** 1 School of Electronic Science and Engineering, Nanjing University, Nanjing, 210093 China; 2 Department of Electronic Materials Engineering, Research School of Physics and Engineering, The Australian National University, Canberra, ACT 2601 Australia; 3 School of Information Engineering, Wuhan University of Technology, Wuhan, 430070 China; 4 Collaborative Innovation Center of Advanced Microstructures, Nanjing University, Nanjing, 210093 China; 5 Collaborative Innovation Center of Solid-State Lighting and Energy-Saving Electronics, Nanjing University, Nanjing, 210093 China; 6 School of Physics and Engineering, Xingyi Normal University for Nationalities, Xingyi, 562400 China

## Abstract

Intermediate band solar cells (IBSCs) are conceptual and promising for next generation high efficiency photovoltaic devices, whereas, IB impact on the cell performance is still marginal due to the weak absorption of IB states. Here a rational design of a hybrid structure composed of ZnTe:O/ZnO core-shell nanowires (NWs) with Al bowtie nanoantennas is demonstrated to exhibit strong ability in tuning and enhancing broadband light response. The optimized nanowire dimensions enable absorption enhancement by engineering leaky-mode dielectric resonances. It maximizes the overlap of the absorption spectrum and the optical transitions in ZnTe:O intermediate-band (IB) photovoltaic materials, as verified by the enhanced photoresponse especially for IB states in an individual nanowire device. Furthermore, by integrating Al bowtie antennas, the enhanced exciton-plasmon coupling enables the notable improvement in the absorption of ZnTe:O/ZnO core-shell single NW, which was demonstrated by the profound enhancement of photoluminescence and resonant Raman scattering. The marriage of dielectric and metallic resonance effects in subwavelength-scale nanowires opens up new avenues for overcoming the poor absorption of sub-gap photons by IB states in ZnTe:O to achieve high-efficiency IBSCs.

## Introduction

Thanks to the strong light-matter interaction, one dimensional semiconductor nanowires (NWs) have opened new avenues in photonics and solar energy harvesting, and provided an extraordinary platform for nano-optoelectronic applications, including lasers, optical switches^1^, photodetectors^2^ and solar cells^3^. The nanowire geometry is particularly interesting as it allows for excellent charge collective extraction and a large optical cross section^4^. By controlling the dimension and morphology, NWs can be synthesized to support distinct dielectric resonances with low optical loss, enabling the manipulation of the optical properties at the deep subwavelength-scale^5, 6^. Meanwhile, various metallic nanostructures have shown unparalleled ability to modify the light response of nanoscale objects through a plasmonic resonant coupling between excitation and metal structures^7^. Especially when light interacts with optical antennas, surface plasmons are generated due to the coupling between adjacent nanoscale resonators. Therefore, semiconductor NWs with high photon confinement using both dielectric and metallic resonance are the promising building blocks for future nanoscale optoelectronic devices with dramatically reduced energy consumption or extraordinary energy conversion efficiency.

For the host NW dielectric material, ZnTe semiconductor has desirable properties for photovoltaic applications, including a direct bandgap of ∼2.25 eV, large absorption coefficient and high refractive index^8^. In particular, oxygen-doped ZnTe (ZnTe:O) exhibits a distinct sub-bandgap emission and photoresponse around 1.8 eV due to oxygen-bounded excitonic absorption^9, 10^. It has been regarded as one of promising materials for intermediate band solar cells which can enable the absorption of low energy photons and reach a detailed balance efficiency limits of 63.2% in theory^11, 12^. The ZnTe:O based intermediate-band solar cell (IBSC) prototypes have been demonstrated but the IB impact on the cell performance is still marginal mainly due to the weak absorption coefficient of IB states^13^. The lack of sufficient light absorption in single NWs due to its small absorption volume also remains challenging for developing efficient concentrated NW solar cells^14^.

In this contribution, we exploit the highly confined dielectric resonance in NWs and plasmonic coupling with integrated bowtie nanoantennas to improve light trapping and boost the broadband absorption of ZnTe:O/ZnO core/shell NWs. The dimension of ZnTe:O/ZnO core/shell NWs are numerically optimized to form the dielectric resonance of leaky modes in visible spectrum region, thus maximizing the overlap between the optical absorption in ZnTe:O and the solar spectrum. The non-traditional plasmonic metal, aluminum (Al) is chosen as the antenna material due to its complementary metal-oxide-semiconductor (CMOS) compatibility, low-cost, sustainability, and mass-productivity^15^. Both theoretical simulation and experimental results are well consistent that, with integration of Al plasmonic bowtie antenna around the ZnTe:O/ZnO core/shell NW, efficient resonant coupling between surface plasmons and dielectric nanowire leads to further enhancement in absorption and radiative emission, particularly for the broadband near 680 nm. The effect of plasmonic antennas on the absorption and its specific resonance behavior are discussed in terms of the evolution of electromagnetic field distribution.

## Structure Design and Fabrication

**Figure 1:**
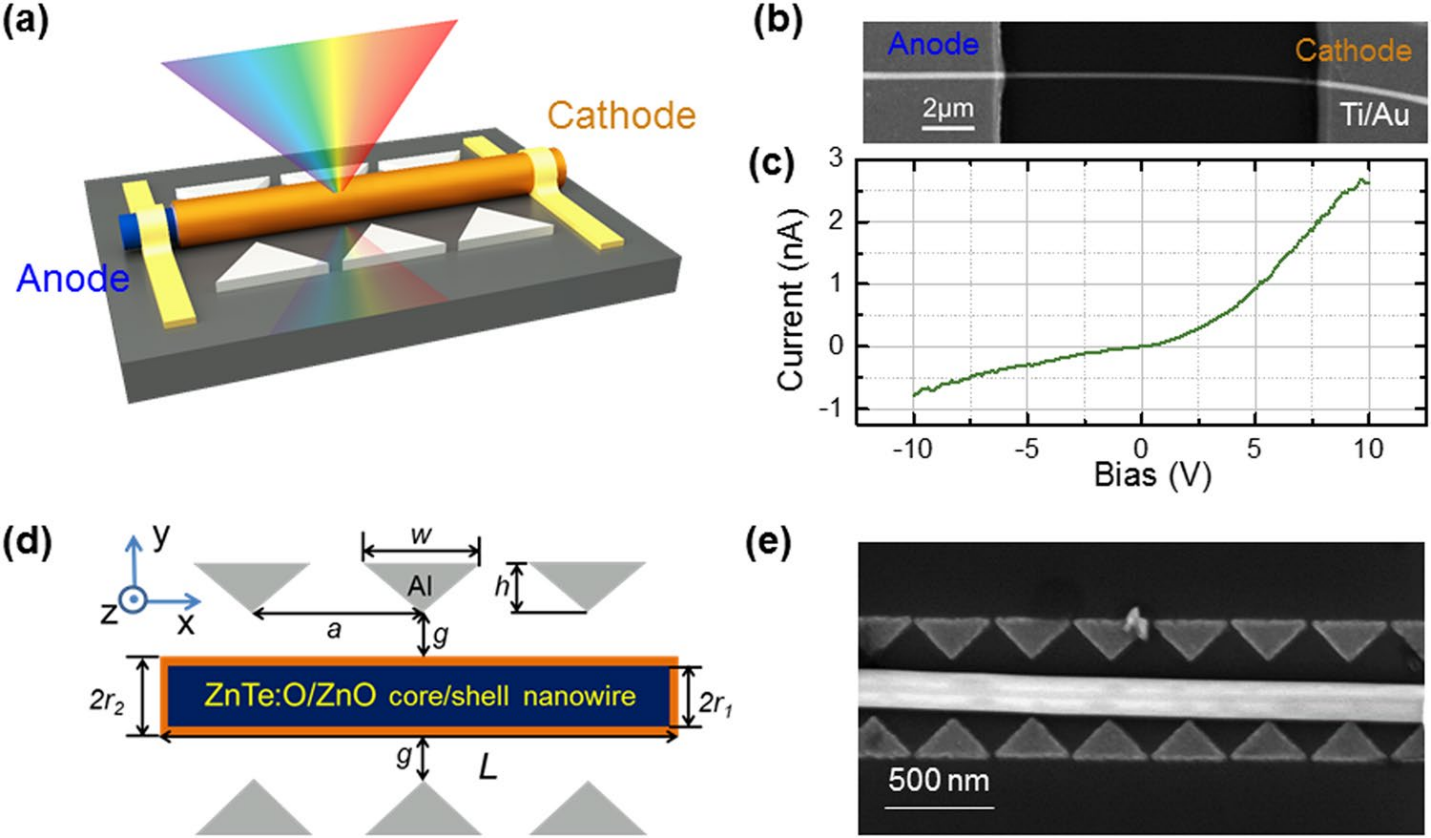
(**a**) Schematic of ZnTe:O/ZnO core/shell NWs coupled to bowtie nanoantennas. (**b**) Scanning electron microscopy (SEM) image and (**c**) the resultant I-V characteristics of a 140-nm-radius ZnTe:O/ZnO core shell nanowire device, (**d**) Top view of the NW with bowtie antennas depicting the various dimensions, (**e**) SEM image of a 140 nm-radius ZnTe:O/ZnO core shell nanowire with aluminum bowtie antennas.

The proposed structure is schematically presented in Fig. 1(a). ZnTe:O/ZnO core/shell NWs are transferred from the grown substrates to a low-index substrate, such as glass or quartz. ZnTe is a typical p-type material, while ZnO is naturally n-type and transparent to visible solar spectrum. By carefully controlling the oxidization of the as-grown ZnTe NWs, ZnTe:O/ZnO core-shell structures have been produced, where ZnTe:O NW core is surrounded by a ZnO shell, forming a p-n heterojunction perpendicular to the growth direction^16^. Then, the individual ZnTe:O/ZnO core-shell nanowire device were fabricated. The details of fabrication processes are described in Methods part. Figure 1(b) shows the SEM image of the individual ZnTe:O/ZnO core-shell nanowire device and a typical rectifying characteristics in Fig. 1(c) verify the formation of p-n junction. Simulation is carried out with a goal of engineering the resonances in ZnTe:O/ZnO core/shell NWs to maximize the overlap of absorption and solar spectra. Al bowtie antenna pairs with a 90° apex angle, 280 nm thick and various arm lengths and numbers are designed surrounding the NWs. As shown by the top-view of the schematic in Fig. 1(d), the antenna consists of various pairs (here showing triple-pairs) of two opposing tip-to-tip Al triangles (width *w*, height *h*, period *a*) on both sides of the single core/shell NW (ZnTe:O radius *r*_1_, ZnTe:O/ZnO radius *r*_2_ = *r*_1_ + 5 nm, length *L*), with the distance *g* from the triangle tip to the NW. After numerical simulation, one-dimensional Al bowtie antenna arrays with optimal dimensions have been fabricated on a Si substrate and the transferred ZnTe:O/ZnO single nanowire is sandwiched in a coupled antenna as shown in Fig. 1(e).

Dielectric resonances with advantage of low optical loss in subwavelength-scale semiconductor NWs are sensitive to NW dimensions and geometry, and thus with optimal dimensions are essential^17^. Based on classical waveguide theory^18^, the NW can be viewed as an ultimately scaled-down version of a microcylinder resonator with radius *r*. By shrinking the NW dimensions, the resonance modes become more leaky, and can interact more effectively with the outside media^19^. In the case of normal-incidence illumination (the wave vectors along the cylindrical axis is zero) on the NW with an infinite length in vacuum (the refractive index in air, *n*_0_ = 1), the excitation conditions can be simply split for purely transverse-magnetic (TM) modes with the magnetic field normal to the NW axis and transverse-electric (TE) modes with the electric field normal to the NW axis^19^. For the NW with a finite length, the electromagnetic field of the incident light suffers diffraction at the two ends of NW and introduces a longitudinal wavevector along the NW. If the NW length satisfies certain conditions, longitudinal-field Fabry-Perot (F-P) resonance will be generated and leads to hybrid modes through interaction with transverse radial resonances^20^.

## Results

**Figure 2:**
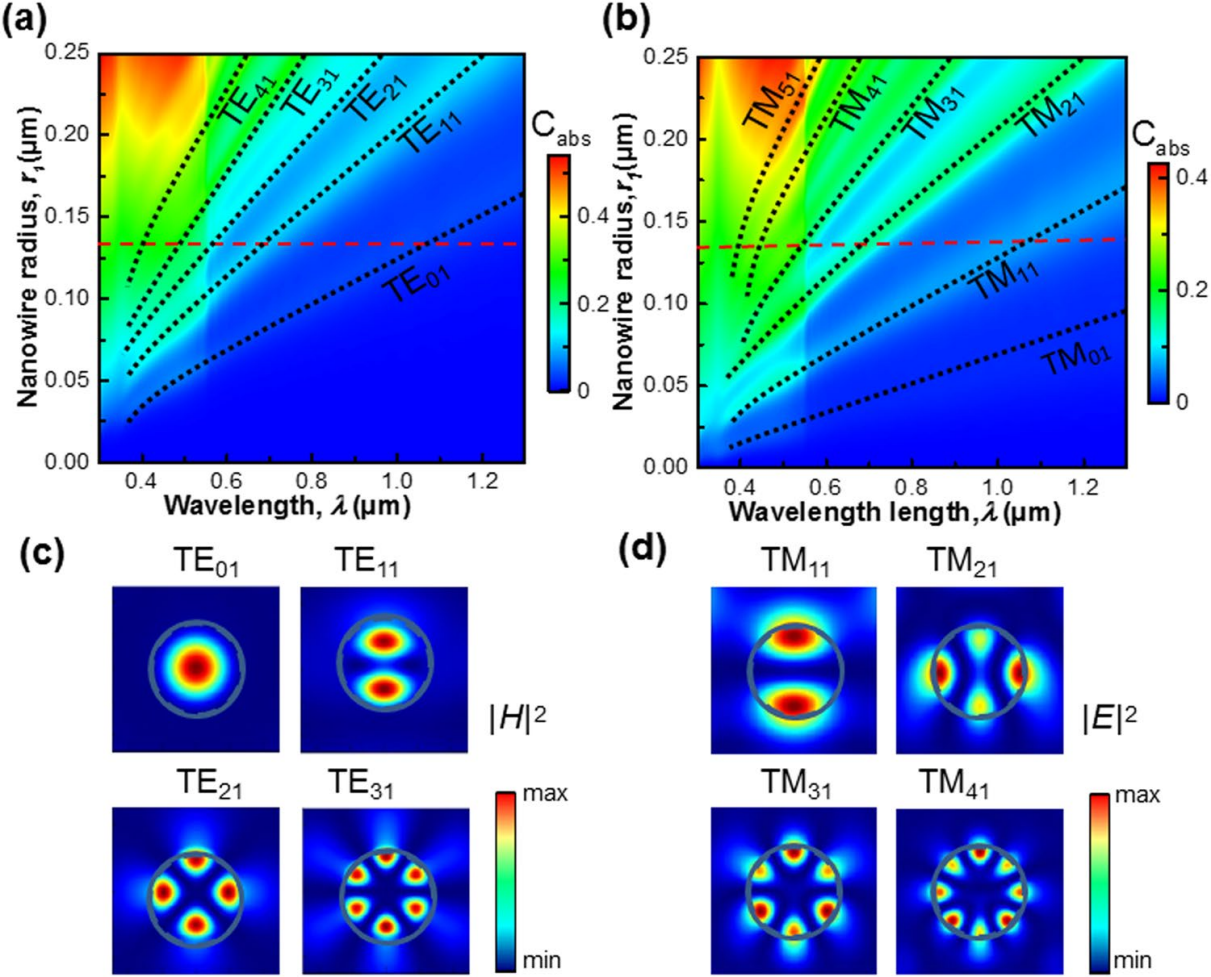
Graphs illustrating the tunability of the absorption resonance peak for single NW. Two-dimensional plot of calculated absorption cross section as a function of wavelength and radius of the NW for TE (**a**) and TM (**b**) modes. The dashed black lines represent the different leaky modes at the wavelength between 300 and 1300 nm. (**c**) The configuration of the magnetic field intensity for typical TE leaky modes. (**d**) The configuration of the electric field intensity for typical TM leaky modes. The blue solid circles and dashed circles represent the ZnTe/ZnO interface and ZnO/air interface, respectively.

In terms of the well-known Mie theory, we perform two-dimensional (2D) FDTD calculation to evaluate the absorption cross sections in both polarizations, assuming an infinite NW length and taking the wavelength-dependent refractive index and substrate influence into account. Figure 2(a) and (b) illustrate the two-dimensional (2D) plot of the absorption cross section versus wavelength and nanowire radius for a ZnTe:O/ZnO core/shell NW under TE and TM incidence, respectively. A sharp drop of absorption cross section at the wavelength of 550 nm is observed regardless of the changes in NW radius, which is due to the low absorption coefficient of ZnTe below its band gap (2.25 eV). Nevertheless, the enhancement of absorption cross sections related to the various transverse electric (TE_*m*l_) and transverse magnetic (TM_*m*l_) resonance modes are clearly displayed, where *m* and *l* represent the azimuthal mode number and radial order of the resonances, respectively. The maxima guided by dark dotted lines in Fig. 2(a) and (b) indicate that the resonances occur for fixed values of *nkr*_1_ that satisfy the resonance conditions, where *n* is the refractive index of nanowire, *k* is the free space wavevector^19^. The resonant wavelengths tend to shift approximately linearly with increasing radius. Note that in cylindrical coordinates, except for the fundamental mode TM_01_, higher order TM and TE modes are approximately degenerate, e.g., TM_11_/TE_01_, TM_21_/TE_11_, TE_31_/TM_41_.By changing the NW radius/diameter, one can tune the resonance wavelength and engineer the desired absorption spectra for specific applications. A key strategy to enhance the absorption efficiency is to increase the number of supported resonances in the NW. As indicated by the red dashed lines in Fig. 2(a) and (b), for a single ZnTe:O/ZnO core-shell NW with a specific core radius of *r*_1_ = 135 nm, multiple dielectric resonances occur 465, 550 and 680 nm as the degenerate leaky waveguide modes of TE_11_/TM_21_, TE_21_/TM_31_ and TE_31_/TM_41_, respectively. If taking the NW length effect into account, the slight variation of resonance wavelength particularly for TE_11_ (680 nm) modes is observed due to the occurrence of longitudinal F-P resonance along the NW axis, as shown in Fig. S1 of Supplementary Materials. It is also reported that the spectral resonance of higher order modes has minimal dependence on angle of illumination if the nanowire length is much larger than the ratio of the supported resonance wavelength to refractive index. Thus, considering the achievable length in growth experiments, we choose *L* = 1.5 μm in this work.

To obtain further insight on the nature of these supported resonance modes, spatial distribution of the near-field intensity for TE and TM azimuthal polarizations are analyzed and presented in Fig. 2(c) and (d), respectively. For TE polarization, the near-field maps in *yz* plane exhibit magnetic multipoles at wavelengths of 1050, 680, 550, and 465 nm that are identified as TE_01_, TE_11_, TE_21_, and TE_31_ modes, respectively. The slight radial asymmetry of the near-field intensity is observed due to substrate effect. The magnetic field for the fundamental TE mode (TE_01_) is highly confined within the NW core, indicating strong light trapping in a whispering-gallery mode configuration^20^. The high field intensities inside the structure can directly contribute to enhanced absorption. The electromagnetic field extends few nanometers out of the nanowire for higher order TE modes, and thus the modes are referred to as leaky-mode resonances which can efficiently interact with surrounding electromagnetic field and improve the radiative loss of core material^19^. In comparison, the spatial distribution of electric field intensity for TM modes (in Fig. 2(d)) shows a more “spread out” effect and is expected to result in enhanced coupling between incident light and leaky modes in the structure. Moreover, it is worthy to note that the surrounding ZnO shell as a non-absorbing material for visible light is expected to boost the solar absorption in the ZnTe:O core by creating new leaky modes and increasing the radiative loss^21^, as well demonstrated by the enhanced field intensity in the ZnTe:O core nanowire in Fig. S2 of Supplementary Information.

**Figure 3:**
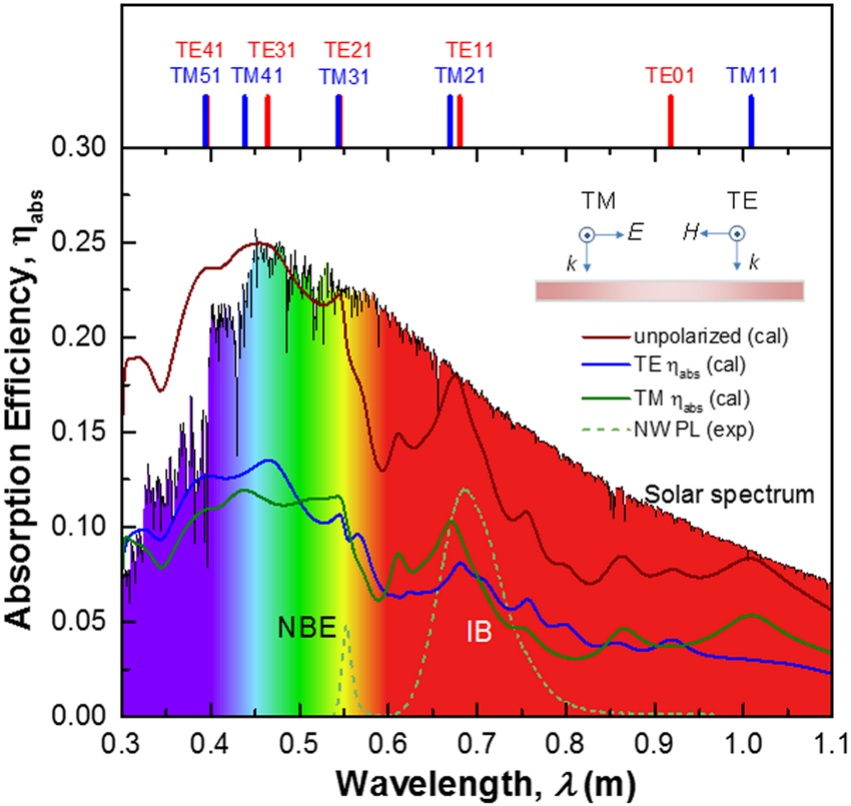
Absorption efficiency spectra of ZnTe:O/ZnO core-shell nanowire with an ZnTe:O core radius of 135 nm calculated using linearly polarized TE (blue), TM (green) and unpolarized light (brown). The inset illustrates the illumination geometry for the TE and TM polarizations. The typical photoluminescence of ZnTe:O nanowires and the solar spectrum are also shown to visualize their correlation with the absorption spectra. The locations of the calculated resonance modes are indicated at the top of the figure.

Next, we examine the role of these leaky-mode resonances in the absorption capability of NWs. The calculated *η*_*abs*_ spectra from a ZnTe:O/ZnO core-shell NW with an inner radius of 135 nm under TE and TM illumination are shown in Fig. 3, together with the AM1.5 solar spectrum and experimental photoluminescence of bare ZnTe NW for reference. The individual spectra exhibit several distinct absorption peaks, well correlated to the occurrence of specific TE or TM leaky resonance modes, as indicated with ticks at the top of Fig. 3. Due to the combination effect of 1D longitudinal and radial transverse components, the resonance of TM modes shifts to higher energy with respect to TE modes, and thus degeneracies of TE and TM modes are broken^20^. The dramatic drop of the absorption efficiency at 550 nm is due to the low absorption coefficient below the bandgap of ZnTe. For ZnTe:O intermediate band solar cells, the key strategy to increase the overall efficiency is to improve the capability of absorbing sub-bandgap energy photons via intermediate states and contributing to the photocurrent. As shown by the photoluminescence of ZnTe:O/ZnO NW, besides the near-band edge emission at 550 nm, the dominant emission near 680 nm corresponds to the optical transition from the intermediate band (IB) in ZnTe:O to the VB, denoted as IB band^9, 10^. Despite the low absorption coefficient *α* of about 10^3^ cm^−1^ for ZnTe used in the calculation, the absorption efficiency for IB near 680 nm under unpolarized light illumination can be boosted to 0.18. Given the higher *α* of 10^4^ cm^−1^ for the VB-IB transition in ZnTe:O^22^, the IB absorption efficiency can be further enhanced. The enhanced sub-bandgap photoresponse has been experimentally demonstrated in the fabricated ZnTe:O/ZnO core-shell single nanowire device (see Fig. S3). A distinct photovoltaic effect was observed with a short-circuit current density (40 mA/cm^2^) and open-circuit voltage (0.61 V). We aware that the obvious difference between the calculated absorption efficiency and the measured photoresponse lies in the spectral region above the bandgap of ZnTe, which may be related to the deviation of geometry/dimension and material quality of nanowires. Nevertheless, the sub-bandgap photoresponse around 680 nm has been enhanced greatly, well consistent with the spectral position of the predicted leaky mode resonances. It implies that dielectric resonance in ZnTe:O core-shell nanowire indeed improves the absorption mediated by intermediate band states.

The quantitative difference in absorption efficiency for TE and TM modes is mainly due to the different matching of radiative loss and absorption loss rates. To understand this behavior, we turn to coupled leaky mode theory, an intuitive model developed by Yu and Cao^23^. It shows that the absorption efficiency *η*_*abs*_ for any resonance $({N}_{real}=nk{r}_{1})$ mode in 1D NW is dictated by ${q}_{abs}{q}_{rad}/{({q}_{abs}+{q}_{rad})}^{2}$, where *q*_*abs*_ and *q*_*rad*_ are defined as the intrinsic absorption loss and radiative loss, respectively^23^. Extensive studies have pointed out that, given the fixed intrinsic absorption loss, the absorption efficiency can be maximized by tuning radiative loss equal to absorption loss. This condition is known as critical coupling, for which the phase and amplitude of the incident and scattered waves are matched to yield complete destructive interference^23–25^. Thus, under unpolarized light illumination, the simultaneous enhancement of absorption efficiency in multiple resonance modes match the solar spectrum very well, which indicates that utilizing dielectric resonance in nanowires could be an effective way to improve the conversion efficiency of the ZnTe:O intermediate band solar cell devices.

**Figure 4:**
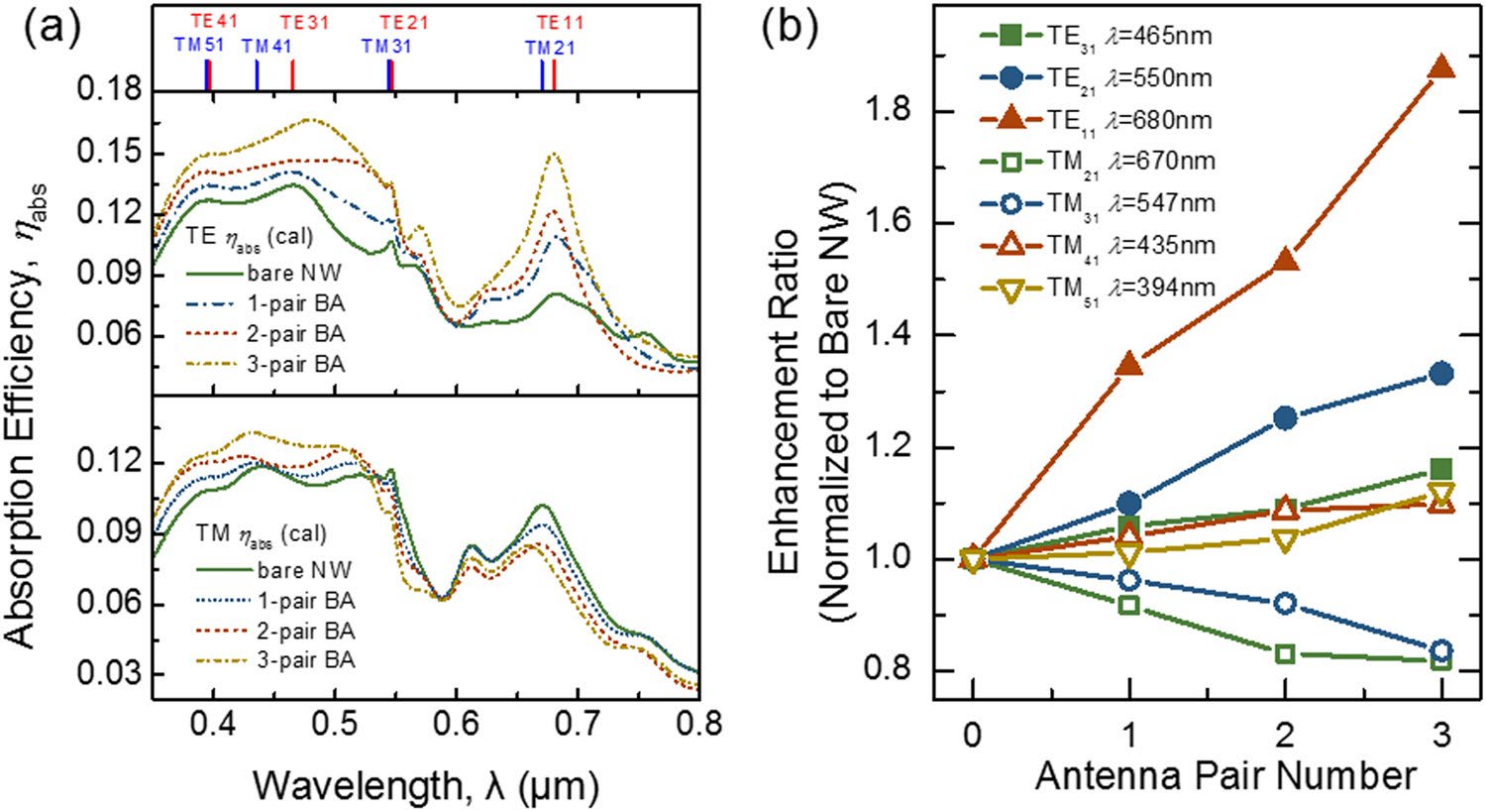
Absorption efficiency with the optimized parameters of the designed device. (**a**) Calculated absorption efficiency for the TE modes and TM modes. The locations of resonance modes are indicated at the top of the figure. (**b**) Absorption enhancement for TE and TM modes with respect to bare nanowire for different number of antenna pairs.

Besides the inherent dielectric resonance, the field enhancement within nanowires can often be further improved by combining it with a complementary metallic plasmonic structure^6^. To achieve this, the Al bowtie antennas are placed in close proximity of a ZnTe:O/ZnO core-shell single NW. By calculating the absorption efficiency using FDTD method, the geometric dimension of the bowtie antenna can be optimized as *w* = 400 nm and *h* = 200 nm, to maximize the energy concentration inside the NW (see Supplementary Fig. S4). The thickness of the antenna is fixed at 280 nm, which is same with NW diameter. For TE-polarization light incidence (with electric field perpendicular to the cylinder axis), the bowtie antenna can provide a strong confinement within its gap^26^. The NW is therefore placed as close as possible to the antenna^6^, i.e., *g* = 0, so that an increase in absorption could be expected in the NWs. To demonstrate the coupling efficiency of bowtie antennas with NWs, we investigate effect the number of bowtie antennas have on the NW. All the bowtie antennas have the same structural parameters. The absorption spectra of the NW with different pairs of antennas under TE and TM polarized illumination are shown in Fig. 4(a), together with the result of a bare NW as reference. As summarized in Fig. 4(b), for the TE absorption spectra, when integrating a single bowtie antenna, the absorption efficiency can be enhanced to 1.05, 1.1, and 1.3 times at the characteristic wavelengths of 465, 550 and 680 nm as compared to that of the bare NW. The absorption could be further increased if a dual-bowtie antenna with *a* = 800 nm is used. For a triple-bowtie antenna, the optimized distance, *a*, is 400 nm and the enhancement factor can reach 1.24, 1.3, and 1.9 times at wavelengths of 465, 550, and 680 nm, respectively. It has been proven that in the bowtie nano-resonators, the paired metal triangles build up charges at the apexes, producing collective charge oscillations, known as local surface plasmon (LSP) modes, and inducing very strong resonant fields in their vicinity in comparison to other geometries^27^. As a result, the plasmonic fields are mostly concentrated between the gap or the paired tips of Al bowtie antennas with small optical mode volumes. It suppresses radiative loss in the metal while creating maximum interaction with the sandwiched nanowire to efficiently propagate energy perpendicularly into the absorbing NW resonator. However, surprisingly in the case of TM illumination, the absorption efficiency decreases for the lower order TM modes (TM_21_) with increasing number of antenna pairs while higher order TM modes behave inversely. Such complicated variation behavior is quite different with TE modes, which may be correlated to their differences in the intrinsic absorption loss and the coupling directivity of incident light with leaky modes in the ZnTe:O NW.

**Figure 5:**
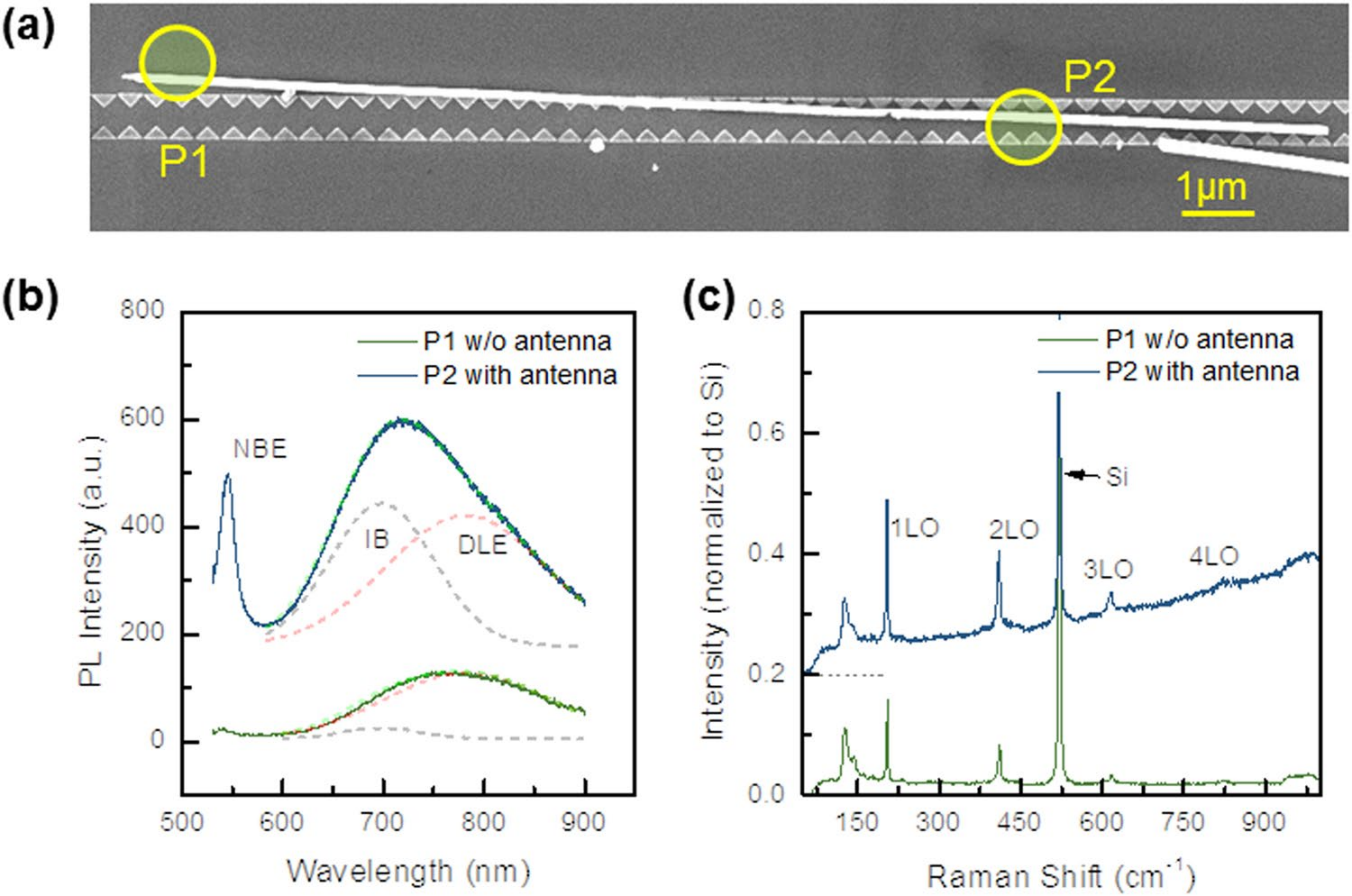
(**a**) SEM image of a 140-nm-radius ZnTe:O/ZnO core shell nanowire integrated with one-dimensional Al antenna array; (**b**) Micro-photoluminescence and (**c**) resonant Raman scattering from nanowire parts located within or outside of the coupled Al bowtie antenna array.

To investigate the modulation of optical properties induced by LSP modes, detailed spectroscopic characterization including photoluminescence and resonant Raman scattering were performed on the individual core-shell nanowire within or outside of the coupled bowtie antenna array as shown in Fig. 5. The PL spectrum from the nanowire outside of bowtie antenna (marked as P1) exhibits a weak near-band edge (NBE) emission at 2.25 eV and a broad deep level emission at 1.6 eV induced by defective stacking faults in the as-grown ZnTe nanowire^10^. In comparison, for the sandwiched part of the same nanowire (P2 in Fig. 5(a)), the NBE emission is dramatically enhanced, as well as the shape and intensity of the broad deep level emission is modulated. By careful Gaussian fitting illustrated in Fig. 5(b), the emission at 1.8 eV related to intermediate band states is greatly enhanced. More clear enhancement feature has been illustrated by the dependence of integrated PL intensity on the incident laser power density (see Fig. S5). The enhanced emission locations at 550 and 690 nm are exactly aligned with the spectral positions of the predicted TM_31_/TE_21_ and TM_21_/TE_11_ degenerated leaky resonance modes, respectively. As the tested single nanowire is quite uniform, it is reasonable to conclude that the distinguished PL enhancement is a consequence of the strong interplay of dielectric and plasmonic resonances. Another direct evidence of efficient exciton-LSP coupling is the observation of profoundly enhanced longitudinal optical-phonons of ZnTe within the coupled Al bowtie antenna as exhibited in the resonant Raman scattering spectra in Fig. 5(c). Typically, the optical transitions for both resonant Raman scattering and photoluminescence involve absorption and re-emission processes. The optical transition rate and density of states is increased as induced by the strong exciton-plasmon coupling and the enhanced dielectric leaky resonance^27, 28^. In terms of Fermi’s golden rules, it is expected to not only enhance the resonant Raman and fluorescence properties but also enable the improvement of excitonic absorption.

## Discussion

**Figure 6:**
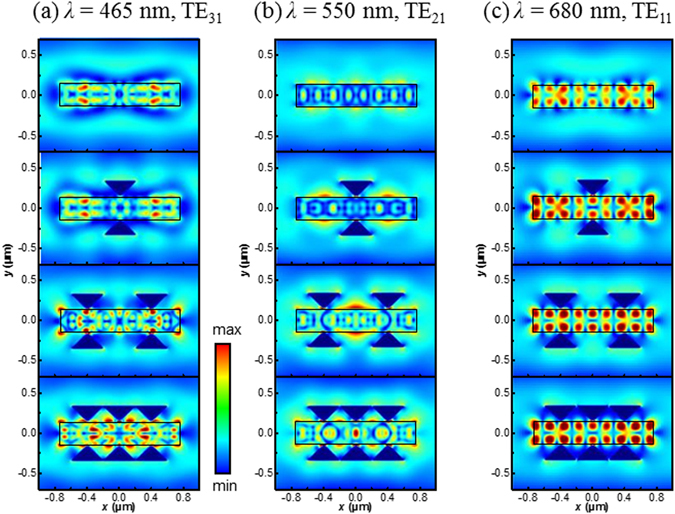
Calculated electric near-field distribution of the NW-antenna system with TE polarized light across the NW plotted in the horizontal plane of the device. (**a**)–(**c**) Show the electric field intensity for the wavelength of 465 nm (TE_31_), 550 nm (TE_21_) and 680 nm (TE_11_) for NW with and without the bowtie antennas, respectively.

**Figure 7:**
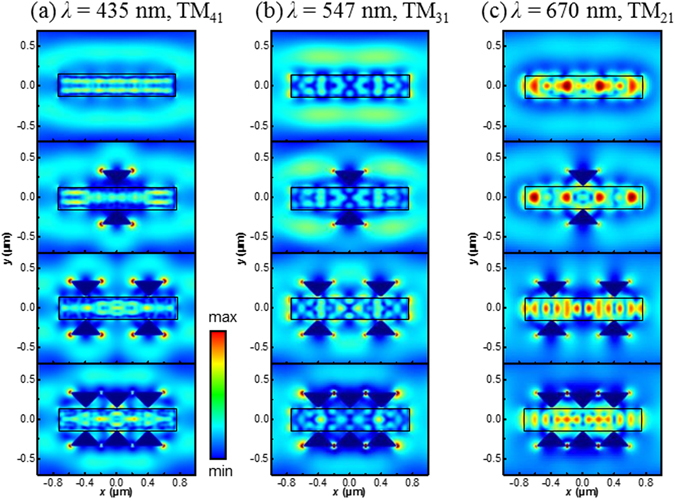
Calculated electric near-field distribution of the NW-antenna systems with TM polarized light across the NW plotted in the horizontal plane of the device. (**a**)–(**c**) Show the electric field intensity for the wavelength of 435 nm (TM_41_), 547 nm (TM_31_) and 670 nm (TM_21_) for NW with and without bowtie antennas, respectively.

To understand the correlation of optical transition and resonance behaviors, we performed 3D FDTD simulations of electric field distribution in the bare NW, as well as a NW integrated with single or multiple pair of bowtie antennas. Figures 6 and 7 plot the spatial distribution of |*E*| in the horizontal *xy* plane of the system for the three characteristic leaky modes under TE or TM incidence, respectively. The absorption coefficient of ZnTe has not been involved in the calculation so as to demonstrate how the light trapping could be improved within the NWs due to the integration of antennas. Upon TE polarized light incidence, Fig. 6 clearly displays that the electromagnetic energy is concentrated and dispersed in the bulk of bare NW due to the well-designed hybrid dielectric resonance. It is evidenced in Figs. 6 and 7 that besides the radial symmetric resonance, the longitudinal F-P resonance along the NWs forms the standing waves. With integration of Al antennas, strong dipolar excitations in the form of localized surface plasmon resonances (LSPRs) are generated at all the antenna tips with dipole moments along the *y*-direction (Fig. 6). The performance of such a type of dipole oscillation strongly depends on the antenna gap (i.e., the values of *g* and *h*). In this work, the optimized parameters *g* = 0 and *h* = 200 nm are chosen for maximizing the light energy injection and concentration into the sandwiched NW. Strong near fields closely couple with the leaky modes and consequently modulate the field distribution profile both inside and outside the dielectric NWs. As stated in ref. 25, the induced high energy confinement within the NWs would be expected to significantly increase the radiative loss *q*_*rad*_, especially for the TE_11_ mode that has a stronger field intensity as compared to TE_21_ and TE_31_ modes. It makes the critical coupling condition to be easily satisfied for the given low *q*_*rad*_ at *λ* = 680 nm and thus exhibits largest enhancement of absorption efficiency as shown in Fig. 4(a). Importantly, with the increase of number of antenna pairs from 1 to 3, the concentration ability of the antenna is enhanced, which is a particularly essential for improving overall absorption capability of NWs, especially for low-energy photons through intermediate band states with limited intrinsic absorption loss.

Under the TM polarized illumination, Fig. 7 displays that the field energy is mainly confined in the bare NW for TM_21_ mode at 670 nm but distributed around the nanowire for TM_31_ and TM_41_ leaky modes. This feature provides obvious evidence for the efficiency differences in the absorption spectra for specific TE and TM modes shown in Fig. 3. With Al bowtie antennas, the dipole LSPRs are mainly generated at the antenna tips on the outer side of the NW with dipole moments along the *x*-direction, more dependent on the width of antenna *w* rather than the antenna height *h* and antenna-NW distance *g*. As compared with the case of TE incidence, such kind of dipolar excitations would be less beneficial for light confinement into the NW and even makes the optical antenna acting as a transmitter rather than a receiver. As shown in Fig. 7, for TM_21_ mode at 670 nm, the field intensity confined in the NW is weakened due to the integration of Al antennas, and more number of bowtie pairs results in weaker light energy concentration in the NW. In this case, the antenna actually acts as an electromagnetic energy sink to extract the absorbing energy from the NW, giving rise to the leakage of radiative loss and thus the decreased absorption efficiency. On the other hand, for TM_31_ and TM_41_ modes, the field intensity confined in the NWs can be slightly enhanced, and increases with the number of bowtie pairs. Such a result is in good agreement with the absorption efficiency improvement at these two high-order modes as shown in the bottom of Fig. 4(a). It should be noted that the wavelength position of resonances can be slightly shifted as compared to 2D simulation results (see Fig. 2), due the finite length effect of the NW in the longitudinal direction. By combining the TE and TM cases, it is found the integration of plasmonic Al bowtie antennas increases the overall radiative loss for all investigated leaky resonance modes and approaching critical coupling condition to improve the absorption efficiency. In particular, regardless of the low intrinsic absorption loss due to limited IB state intensity, the combined dielectric and metallic resonance leads to profound enhancement of absorption efficiency.

In summary, we have designed and demonstrated the multiple-band solar absorption enhancement in ZnTe:O/ZnO core-shell nanowire integrated with Al bowtie plasmonic antennas by the combination of dielectric and metallic resonances. The light absorption of the nanowire core-shell structure is governed by the coupling efficiency between incident light and leaky resonance modes. By engineering these leaky modes, the absorption efficiency related to the transitions of VB-CB and VB-IB are boosted simultaneously. Moreover, through the coupling with bowtie antennas composed of low-cost and CMOS-compatible Al metal with proper dimensional designs, the overall absorption efficiency exhibits a significant additional enhancement with respect to the bare nanowires, owing to efficient energy injection through LSPRs around the sharp tips of the antennas. The enhance absorption especially for intermediate band states has been verified by the enhanced photoluminescence and photoresponse in an individual ZnTe:O/ZnTe core shell nanowire device. Our results indicate that it is possible to increase absorption by coupling the metallic antennas induced plasmonic resonances with the dielectric resonances in core-shell NWs. It provides an exciting set of building blocks for various applications which requires high absorption efficiency such as intermediate band solar cells and high-sensitivity photodetectors.

## Methods

### Numerical simulations

Three-dimensional FDTD simulations were performed using a commercial software package (Lumerical FDTD Solutions software). Perfectly-matched-layer boundary conditions are considered carefully to properly define the computational window. Throughout this paper, the permittivity of Al follows the Lorentz-Drude model at all operating wavelengths, and the wavelength dependent absorption coefficient, refractive index of ZnTe, and ZnO materials are obtained from data in refs 8 and 29 (see Supplementary Fig. S6).

The absorption efficiency, *η*_*abs*_ is defined as:^26^

$${\eta }_{abs}=\frac{{C}_{abs}}{{C}_{geo}}=\frac{{k}_{{\rm{0}}}}{2{r}_{{\rm{1}}}{E}_{{\rm{0}}}^{{\rm{2}}}}{\int }_{{\rm{0}}}^{{\rm{2}}\pi }{\int }_{{\rm{0}}}^{{r}_{{\rm{0}}}}\varepsilon ^{\prime\prime} (r){|E(r,\phi )|}^{{\rm{2}}}rdrd\phi ,$$

where *C*_*abs*_ and *C*_*geo*_ are the absorption and geometric cross sections, respectively, *r*_1_ is the inner radius of core-shell nanowire, ε″ is the imaginary part of the relative permittivity of ZnTe, *E*_0_ and *E* represent the electric field amplitude for incident light and detected light within NW, respectively. In the simulation, plane wave excitation at normal incidence is used, as shown in Fig. 1(a). A non-uniform orthogonal mesh grid is used to reduce the computational costs. The mesh size at the material interfaces is set to be 2 nm which is much smaller than the element sizes and the operating wavelength.

### Experimental Section

The ZnTe nanowires were grown by chemical vapor transport technique at 470 °C on GaSb (100) substrates in a horizontal furnace tube. Controlled oxidization processes were performed on the as-grown ZnTe nanowires at 250 °C at oxygen ambient with an atmosphere pressure for four hours. Oxygen diffusion leads to the formation of n-ZnO shell and p-type ZnTe:O inner cores. To fabricate individual nanowire p-n junction, the prepared core-shell nanowires with length over 10 μm were mechanically transferred onto the SiO_2_/Si substrates. Then Ti (10 nm)/Au (50 nm) contact was made on ZnO outer shell of one end of nanowire by the standard photolithography and electron-beam evaporation process. Subsequently, the second lithography process was made, just leaving another end of nanowire to be exposed, followed by the wet etching of ZnO outer shell in diluted HCl solution and electron-beam evaporation of Ti/Au metals to form anode. One dimensional Al plasmonic bowtie antenna arrays (50 nm Al in thickness) were fabricated on bare silicon substrates by processes of electron beam lithography, electron-beam evaporation and liftoff. Then the prepared core-shell nanowires were mechanically transferred onto the templates with patterns of Al bowtie antennas. The morphology was characterized by field emission scanning electron microscopy (FESEM, Zeiss) operating at 5 kV. Micro-photoluminescence and resonant Raman scattering spectra were recorded at room temperature using a Micro-Raman spectrometer with a Horiba JY T64000 spectrometer system in a backscattering configuration with a 514 nm Ar^+^ laser as the excitation source. Spectral photocurrent measurements were carried out with a 500 W xenon-arc lamp coupled to a monochromator (Model iHR320) and the incident power density was calibrated by a Si reference photodiode.

## Electronic supplementary material

Supplementary information
