# Supplementary material for: Extreme absorption enhancement in ZnTe:O/ZnO intermediate band core-shell nanowires by interplay of dielectric resonance and plasmonic bowtie nanoantennas
Source: Sci Rep. 2017 Aug 8;7:7503. doi: 10.1038/s41598-017-07970-7 (PMC5548811; doi:10.1038/s41598-017-07970-7)
Supplement: Supplementary file 1 — Supplementary information [file 41598_2017_7970_MOESM1_ESM.pdf]

Supplementary Information

Extreme absorption enhancement in ZnTe:O/ZnO intermediate band core-shell nanowires by interplay of dielectric resonance and plasmonic bowtie nanoantennas

Kui-Ying Nie[1,6], Jing Li[1], Xuanhu Chen[1], Yang Xu[1], Xuecou Tu[1], Fang-Fang Ren[1,2,4*], Qingguo Du[3*], Lan Fu [2], Lin Kang[1], Kun Tang [1], Shulin Gu,[1] Rong Zhang[1], Peiheng Wu, [1] Youdou Zheng[1], Hark Hoe Tan[2], Chennupati Jagadish[2], Jiandong Ye[1,2,5*]

[1] *School of Electronic Science and Engineering, Nanjing University, Nanjing 210093, China*

[2] *Department of Electronic Materials Engineering, Research School of Physics and Engineering, The Australian National University, Canberra ACT 2601, Australia*

[3] *School of Information Engineering, Wuhan University of Technology, Wuhan 430070, China*

[4] *Collaborative Innovation Center of Advanced Microstructures, Nanjing University, Nanjing 210093, China*

[5] *Collaborative Innovation Center of Solid-State Lighting and Energy-Saving Electronics, Nanjing University, Nanjing 210093, China*

[6] *School of Physics and Engineering, Xingyi Normal University for Nationalities, Xingyi 562400, China*

1. NW length effect on dielectric resonance modes

Here we investigate the effect of the nanowire length, L, on the resonance wavelength of different leaky modes in the absorption efficiency spectra by 3D FDTD simulations. For instance, Fig. S1 exhibits that, with the value of L increasing from 1 to 2 μm, the wavelength positions of TE_{11} (680 nm) exhibit a periodic feature and the deviation in magnitude of wavelength becomes smaller with increasing NW length. This behavior is related to the Fabry-Perot (F-P) resonance. For the NW with a finite length, the diffraction the electromagnetic wave of incident light suffer diffraction at two end of NW and introduce longitudinal wavevector along NW. If the NW length satisfies certain conditions, longitudinal-field Fabry-Perot (F-P) resonance will be generated and leads to the hybrid modes by combining with the transverse radial resonances. However, for the higher order modes TE_{21} (550cm) and TE_{31} (465nm), the corresponding resonant wavelength is equal to or shorter than the cut-off

*Correspondence and requests for materials should be addressed to F.-F.R. (ffren@nju.edu.cn), Q. G. D (qingguo.du@whut.edu.cn) and J. D. Y. (yejd@nju.edu.cn)

wavelength of ZnTe:O materials (550cm), and thus, the diffracted incident light along the NW axis would be absorbed in a penetrated length at two ends. As a result, the Fabry-Perot (F-P) resonance would not occur for high order modes, and the longitudinal contribution is negligible, leading to the unchanged spectral position of resonances as shown in Fig. S1. Taking the achievable length in growth experiments and also the optimized absorption efficiency, we choose L = 1.5 μm in this work.

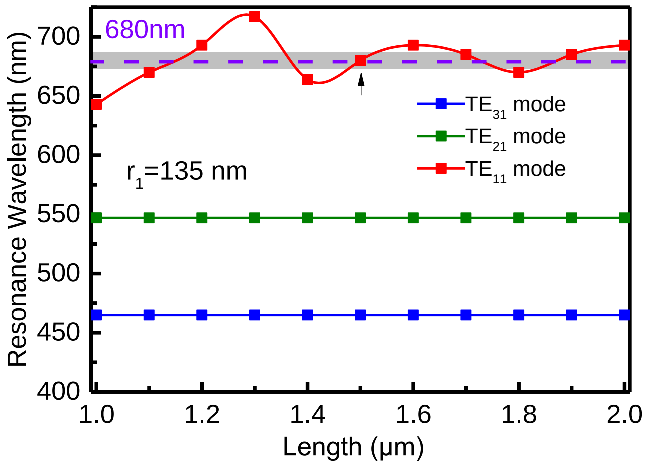

Fig. S1. The resonance wavelength of the different leaky modes (TE_{11}, TE_{21}, and TE_{31}) in ZnTe:O/ZnO nanowire as a function of the length of the nanowire.

2. ZnO Shell effect on field confinement

Heterostructuring with non-absorbing materials is reported as a promising strategy to engineer of the radiative loss of leaky modes and enhance the solar absorption [1-3]. In this work, the ZnO shell as a non-absorbing material is transparent to visible solar light and has a lower refractive index, which helps to enhance the solar absorption when forming heterostructures with absorbing core of ZnTe:O. We now examine the improved absorption enhancement of heterostructures from the perspective of leaky modes. Fig. S2 shows the distribution of magnetic field for TE_{11} mode (λ =680nm) with bare ZnTe:O core nanowire (a) and with ZnTe:O/ZnO core/shell nanowire (b). Based on the conventional waveguide theory, the field in the NW with a coated dielectric shell is obviously more concentrated as

compared to the bare ZnTe:O. The maxima of energy intensity for TE_{11} mode changes by more than one order of magnitude from 0.03 in the pure structure to 1.5 in the full-coated structure. As stated in Ref. 1 by coating a dielectric shell may actually create new leaky modes, and the improved absorption enhancement mainly results from the increase in the radiative loss original modes in the ZnTe:O core. Thus, the oxidization of ZnTe to form ZnO can engineer the radiative loss of leaky modes, which can essentially accounts for all the improvements in the solar absorption of heterostructures [2-3].

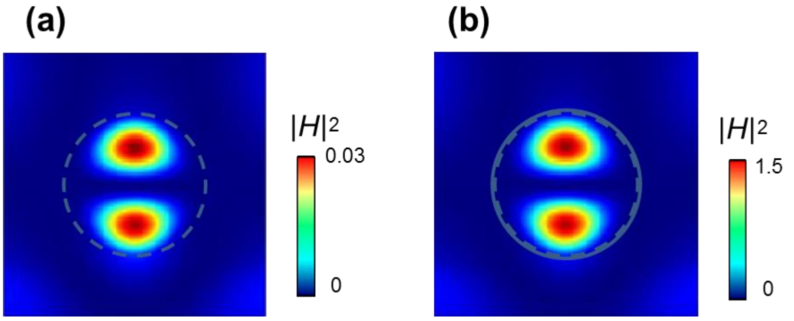

Fig. S2. The configuration of the magnetic field intensity for TE_{11} mode (λ =680nm) with bare ZnTe:O core nanowire (a) and with ZnTe:O/ZnO core/shell nanowire (b) based on conventional waveguide theory. The dash line in (a) indicates the location of ZnTe:O/air interface. The blue solid and dash circle in (b) indicates the location of ZnO/air interface ZnTe:O/ZnO core/shell interface respectively.

3. Photoresponse of ZnTe:O/ZnO core-shell nanowire device

Figure.S3 shows the current-voltage characteristics of ZnTe:O/ZnO core-shell p-n junction and its spectral photoresponse. It was found that Ti/Au electrodes forms good Ohmic contacts on n-ZnO and p-ZnTe:O. By applying bias with ZnO as a cathode and ZnTe:O as an anode, the device exhibits a typical rectifying characteristic, verifying the formation of p-n heterojunction perpendicular to the growth direction. Distinct photovoltaic effect was also observed with a short-circuit current density (40 mA/cm^2) and open-circuit voltage (0.61 V). It is noted that the open-circuit voltage is much lower than the that of sub-gap VB-IB transition due to the presence of defects such as stacking faults. Even so, the device performance is better than the reported ZnTe:O/ZnO nanowire solar cell [4]. Fig. S3 (c)

illustrated that the sub-bandgap photoresponse around 680 nm has been enhanced greatly, well consistent with the spectral position of the predicted leaky mode resonances. We aware that the obvious difference between the calculated absorption efficiency and the measured photoresponse lies in the spectral region above the bandgap of ZnTe, which may be related to the deviation of geometry, dimension, material quality and non-ideal p-n junction formation of nanowires.

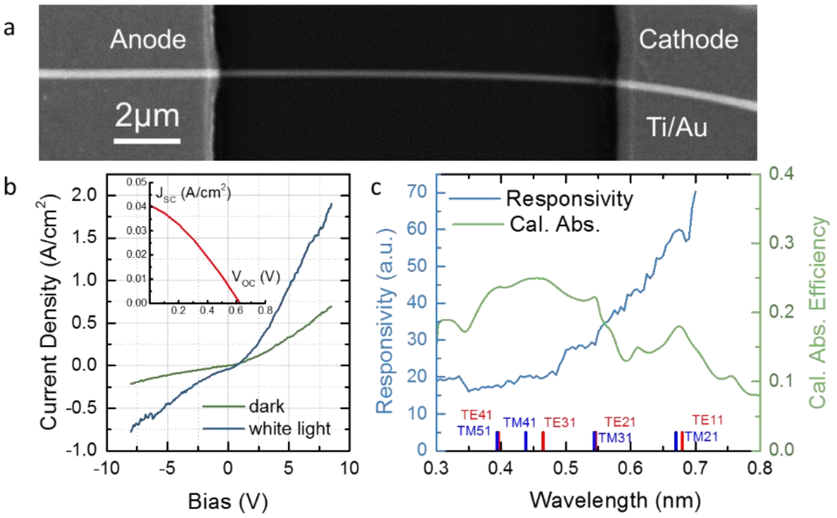

Fig. S3. (a) SEM image of a 140-nm-radius ZnTe:O/ZnO core shell individual nanowire device; (b) Current-voltage characteristics of the device in dark and white light illumination conditions; (c) Spectral photoresponse recorded at the bias of -1V and the calculated absorption efficiency for comparison.

4. Geometrical optimization of Al bowtie antennas

The shape, dimension and spatial distribution of Al bowtie antennas is expected to have influence on the generation of local surface plasmon and the coupling efficiency with the leak modes within the bulk of NWs. The geometrical parameters of Al bowtie antennas are optimized to maximize the absorption efficiency particularly for the intermediate band near 680nm, as shown In Fig. S4 (b)-(e). In all calculations, the doping effect on the NW optical transition properties are not taken into account. Fig. S4 (b) shows that the absorption efficiency near 680nm changes as a function of the height with a fixed width *w*=400nm. As the height increases, the efficiency peak increases in intensity and shifts to

long wavelength near 715nm. Focusing on the intensity evolution near 680 nm, it is found that the case with h= 200nm is optimal. Next, we turn to optimize the width of antenna and find that the absorption efficiency has slight dependence on the width, in other word, less sensitive to the angle of tip adjunct to the nanowire. From the insert of Fig.S4 (c), the width *w*=400nm sounds the best. Thus, in this work, we choose the optimal dimension of w = 400 nm and h = 200 nm in the simulation to maximize the energy concentration inside the NW. Finally, we examine the effect of antenna's distance a on the absorption efficiency. For the case with two pairs antennas integrated, as shown in Fig. S4 (d), the absorption peak near 680nm increases with the increment of distance till a = 800nm and then decreases in intensity as a = 1000nm. Thus, in the design of two pairs antennas, the distance is fixed as a = 800nm. The same variation trends are observed excepted for the optimized condition of a = 400nm for the case in which three pairs of bowtie antennas are integrated, as shown in Fig. S4 (e).

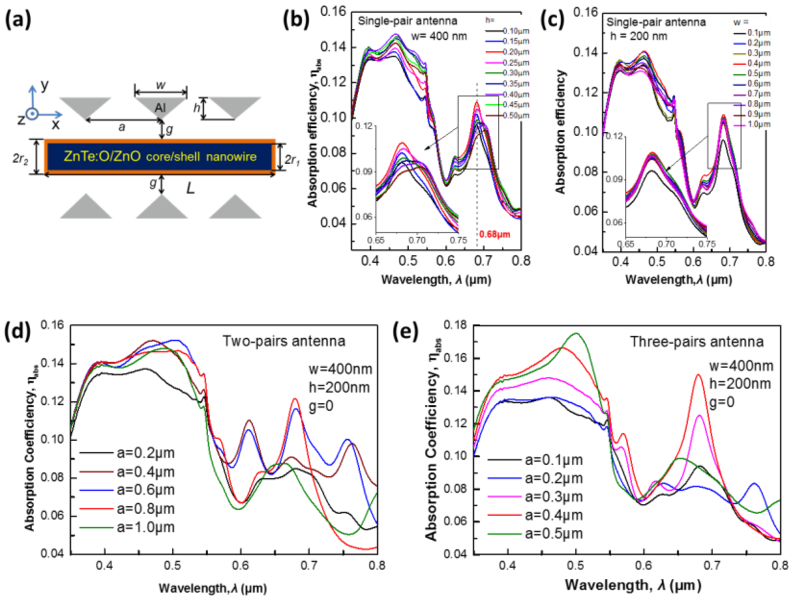

Fig. S4. (a) The top-view of NW/antennas system. The absorption efficiency inside the NW for the different height (b) and width (c) of the antennas. (d) Calculated absorption efficiency inside the NW with two pairs of bowtie antennas for various antennas distance a. (e) Calculated absorption efficiency inside the NW with three pairs of bowtie antenna for various antennas distance a.

5. Power-dependent photoluminescence of ZnTe:O/ZnO core-shell nanowire

Figure S5 shows the photoluminescence spectra under different incident power density of Ar^+ laser (514nm) performed on the individual core-shell nanowire within or outside of the coupled bowtie antenna array. The PL spectrum from the nanowire part outside of bowtie antenna (marked as P1) exhibits a weak near-band exciton (NBE) emission at 2.25 eV and a broad deep level emission at 1.6 eV induced by defective stacking faults in the as-grown ZnTe nanowire. In comparison, for the nanowire part coupled with Al bowtie antenna, both NBE at 2.25 eV and broad band emissions at 1.8 eV are dramatically enhanced. Fig. S5 (c) exhibits the integrated PL intensity as a function of incident power for the NBE emissions. For the same incident power, the NBE emission from the nanowire coupled with Al bowtie antenna are one-order higher in intensity. The increased slope of ln(I)-ln(P) indicates that the optical transition rate of spontaneous emission is increased, which is caused by the strong exciton-plasmon coupling and the enhanced dielectric leaky resonance.

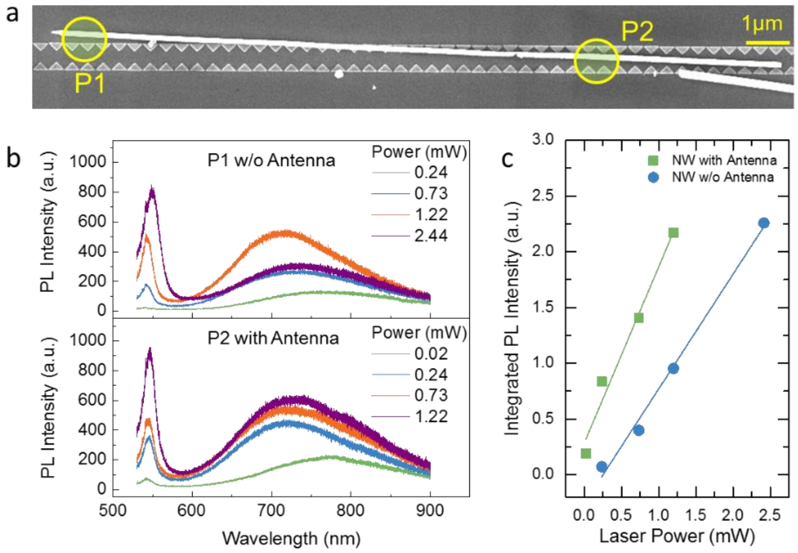

Fig. S5. (a) SEM image of a 140-nm-radius ZnTe:O/ZnO core shell nanowire integrated with one-dimensional Al antenna array; (b) Micro-photoluminescence from nanowire parts located within or outside of the within or outside of the coupled Al bowtie antenna array; (c) Integrated PL intensity as a function of incident laser power density.

6. The refractive index of ZnTe and ZnO

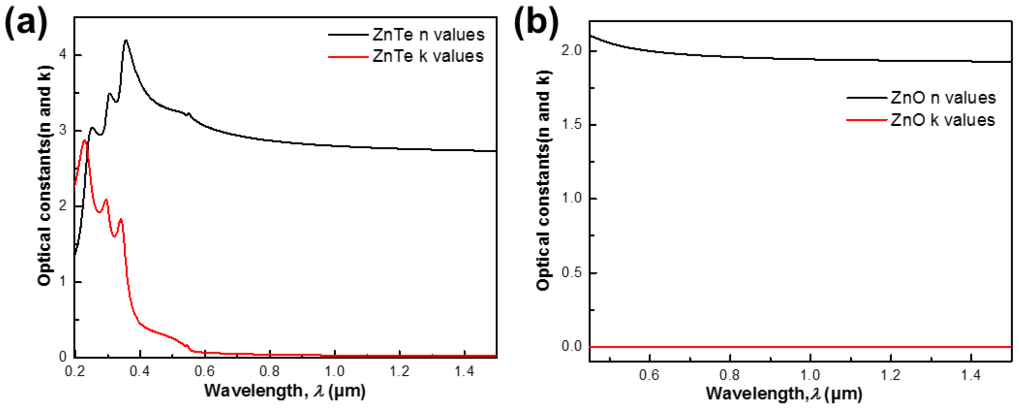

Fig. S6. The wavelength dependent refractive index of ZnTe (a) and ZnO (b) used in this work.

References:

1. Yu Y.; Cao, L., "Leaky Mode Engineering: A General Design Principle for Dielectric Optical Antenna Solar Absorbers", Opt. Commun., 314, 79-85 (2014).

2. M.D. Kelzenberg, et al. "Enhanced absorption and carrier collection in Si wire arrays for photovoltaic application," Nat. Mater. 9, 239-244 (2010).

3. Y. Yao, et al. "Broadband light management using low-Q whispering gallery modes in spherical nanoshells," Nat. Commun. 3, 664 (2012).

4. S. Luo, et al. "Facile in situ synthesis of dendrite-like ZnO/ZnTe core/shell nanorod heterostructures for sensitized solar cells," J. Mater. Chem. C, 4, 4740 (2016).
